# A Hyperstable Aqueous Zinc‐Ion Battery Based on Mo_1.74_CT_z_ MXene

**DOI:** 10.1002/smll.202409122

**Published:** 2025-02-18

**Authors:** Ningjun Chen, Rodrigo Ronchi, Joseph Halim, Per O. Å. Persson, Leiqiang Qin, Johanna Rosen

**Affiliations:** ^1^ Materials Design Division Department of Physics Chemistry, and Biology (IFM) Linköping University Linköping 58183 Sweden; ^2^ Thin Film Physics Division Department of Physics Chemistry, and Biology (IFM) Linköping University Linköping 58183 Sweden

**Keywords:** defects, Mo_1.74_CT_z_, MXene, stability, zinc‐ion battery

## Abstract

The sustainable utilization of natural resources and growing demand for various electronic devices have promoted the development of safe, stable, and rechargeable aqueous zinc‐ion batteries (AZIBs). However, a stable cathode material is crucial for ZIBs in an aqueous electrolyte, since it is more difficult for divalent Zn^2+^ to be reversibly inserted and extracted between active materials than it is for monovalent metal ions. In this work, a tailored multi‐defect MXene, Mo_1.74_CT_z_, of a complete chemical formula of Mo_1.74±0.06_CO_0.95±0.02_(OH)_0.63±0.01_F_0.3±0.03_.0.2±0.05H_2_O_ads_ (Mo_1.74_CT_z_), is assembled as cathode in AZIBs. It achieved 75% capacity retention and nearly 100% Coulombic efficiency even after up to 100 000 cycles as the intrinsic structural stability and many vertical holes of the Mo_1.74_CT_z_ MXene contributed to alleviating the MXene collapse under repeated charge and discharge. Meanwhile, the Mo_1.74_CT_z_‐based AZIBs exhibited good performance with a specific capacity of 200 mAh g^−1^ at a current density of 0.2 A g^−1^, which greatly exceeds previous reports of pure MXene‐based cathodes in AZIBs. This work will aid in finding new solutions for sustainable energy development, which will pave the way for AZIBs as an alternative to lithium‐ion batteries (LIBs) in the future.

## Graphical abstract

A defect‐engineered Mo_1.74_CT_z_ MXene‐based zinc‐ion battery achieves high capacity as well as 75% capacity retention and nearly 100% Coulombic efficiency even after up to 100 000 cycles. The mechanism of hyperstability is revealed to be the intrinsic structural stability and abundant vertical holes of the Mo_1.74_CT_z_ MXene, which contribute to alleviating the collapse of MXene under repeated charge and discharge.

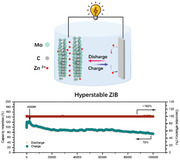

## 1 Introduction

Stable and environmentally friendly rechargeable battery systems with low flammability and high energy density are widely considered portable energy storage devices due to their high energy efficiency, long cycle life, and independence from geographical conditions. Their application scenarios include microgrids, communication equipment, and building backup power supplies in remote areas.^[^ ^1^ ^]^ Currently, commercially produced lithium‐ion batteries (LIBs) are associated with challenges such as a shortage of lithium resources, high cost, and safety hazards of organic electrolytes.^[^ ^2^ ^]^ An ideal rechargeable battery for stationary energy storage should have high security, long cycle life, good environmental friendliness, and low cost.^[^ ^3^ ^]^ In contrast, the energy density is not as much of a concern. Considering the above issues, the rechargeable aqueous battery becomes a promising choice.^[^ ^4^ ^]^ Compared with low‐valence metal ions, multi‐valent metal ions have higher theoretical capacity and safety because they can react with multiple electrons. For example, Al, Mg, and Zn all have higher volumetric and gravimetric capacities.^[^ ^5^ ^]^ Nonetheless, in the aqueous storage system, the Mg anode is strongly passivated, and the Al anode is prone to generate an Al_2_O_3_ film and tends to corrode unevenly. In this respect, zinc‐ion storage has attracted much attention^[^ ^6^ ^]^ because of its many unique advantages. Metallic zinc not only has a theoretical capacity of 820 mAh g^−1^ but also has a redox potential of only −0.76 V versus the standard hydrogen electrode.^[^ ^7^ ^]^ In addition, it has advantages involving abundance, security, cost‐effectiveness, non‐toxic characteristics, and a simple fabrication process.^[^ ^8^ ^]^

Despite these multiple advantages, the development of rechargeable Zn‐ion batteries (ZIBs) has been largely limited by cathode materials.^[^ ^9^ ^]^ The sluggish transport kinetics make divalent Zn^2+^ hard to insert into or extract from the cathode framework due to the large solvation sheath and stronger positive polarity of Zn^2+^.^[^ ^10^ ^]^ Normally, the cathode materials in ZIBs are generally rich in zinc storage sites and should be structurally suitable for the insertion and extraction of Zn^2+^ ions. Materials such as manganese dioxide,^[^ ^7^, ^9^, ^11^ ^]^ Prussian blue,^[^ ^7^, ^12^ ^]^ quinone analogs,^[^ ^13^ ^]^ polyanion metal phosphates,^[^ ^14^ ^]^ and vanadium‐based compounds^[^ ^15^ ^]^ have all been studied as cathode materials for ZIBs. However, these materials suffer from serious problems such as poor electrical conductivity, unwanted phase transitions, material dissolution, and structural collapse,^[^ ^16^ ^]^ which greatly affect the stability of ZIBs—although the mild acid or neutral aqueous electrolyte system can improve the cycling durability of ZIBs.^[^ ^10c^ ^]^ Developing a novel Zn‐ion cathode material with high reversible specific capacity and long cycle life is still a significant challenge.^[^ ^17^ ^]^

Recently, 2D MXenes (such as V_2_CT_z_ and Ti_3_C_2_T_z_) with stable layered structures and controllable layered spacing have become extremely promising candidates as cathodes for ZIBs.^[^ ^18^ ^]^ The general formula of MXene is M_n+1_X_n_T_z_,^[^ ^19^ ^]^ where M is a transition metal (Ti, V, Zr, Nb, Ta, Mo, etc.), X is carbon, nitrogen, or their mixture, and T is the functional groups (─OH, ─F, ─Cl, etc.) typically inherent from the etching process.^[^ ^20^ ^]^ The unique combination of excellent conductivity, hydrophilic performance, special lamellar structure, and unique physical and chemical properties, not only form a skeleton that is conducive to electron/ion transport and promotes electrochemical kinetics but also provides many active sites for ion insertion/extraction or adsorption/desorption.^[^ ^20^, ^21^ ^]^ Moreover, even small defect concentrations can be manipulated and unlock the huge potential of 2D materials without destroying the pristine lattices, benefiting from the high exposure ratio of surface atoms compared to the 3D bulk, also shown for MXenes.^[^ ^22^ ^]^ Furthermore, a reported attainable large interlayer distance of 1.15 to ∼12 nm and tailorable defect structure of MXene may improve the stability and Coulombic efficiency in ZIBs,^[^ ^23^ ^]^ providing open channels for the smooth transport of Zn^2+^ into MXene without causing the collapse of the MXene sheet structure. Therefore, defect‐rich MXene could be an ideal cathode material for long‐lasting and stable ZIBs.

Herein, a tailored Mo_1.74_CT_z_ MXene obtained through defect engineering was realized from etching (Mo_0.87_Cr_0.13_)_2_Ga_2_C, where defects are formed as a result of the dissolution of the sacrificial metal Cr. The MXene delivers a capacity of 200 mA h g^−1^ at a current density of 0.2 A g^−1^ as the cathode for ZIBs. The Mo_1.74_CT_z_//Zn cell exhibits a long‐term cyclability with 75% capacity retention over 100 000 cycles (at a current density of 10 A g^−1^), and the Coulomb efficiency remains close to 100%. Such excellent prolonged cycling stability is mainly due to the intrinsic structural stability and the large number of vertical holes in the Mo_1.74_CT_z_ MXene, which alleviate the MXene collapse under high‐speed Zn‐ion insertion/extraction. Moreover, X‐ray photoelectron spectroscopy (XPS) was used to further explore the reaction mechanism of Mo_1.74_CT_z_ electrodes. During the process from discharge to charge, the intensity of Zn 2p shifts from high to low, and the valence of Mo changes from low to high, indicating that Mo_1.74_CT_z_ MXene has extremely high reversibility in the redox process. This work not only provides a milestone for further understanding the insertion process of Zn ions in MXene‐based ZIBs, but also presents promising hyperstable energy storage with a very long lifespan.

## 2 Results and Discussion

**Figure 1:**
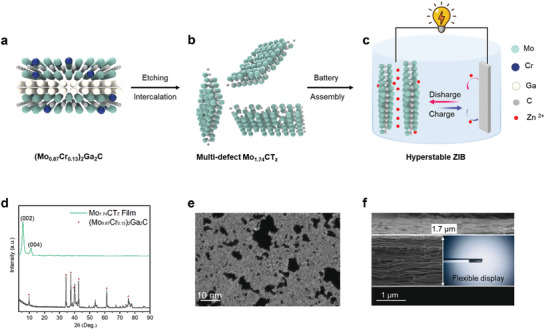
Assembly of hyperstable ZIBs and characterization of Mo_1.74_CT_z_ MXene. a) Precursor (Mo_0.87_Cr_0.13_)_2_Ga_2_C MAX phase. b) Multi‐defect Mo_1.74_CT_z_ MXene nanosheets. c) Assembled hyperstable zinc‐ion battery. d) XRD of (Mo_0.87_Cr_0.13_)_2_Ga_2_C MAX phase and Mo_1.74_CT_z_ MXene. e) High‐resolution transmission electron microscopy (HRTEM) of multi‐defect Mo_1.74_CT_z_ MXene nanosheets. f) Cross‐sectional SEM of a Mo_1.74_CT_z_ MXene film; the inset is an optical image of the flexible film.

**Figure** **1** shows the systematic process going from the precursor (Mo_0.87_Cr_0.13_)_2_Ga_2_C MAX phase to multi‐defect Mo_1.74_CT_z_ MXene nanosheets and the full‐cell AZIBs. A schematic atomic structure of the 221 type MAX phase is shown in Figure 1a. To improve the inherently low vertical ion pass rate of MXene nanosheets, Ronchi et al. purposefully solid‐solubilized Cr atoms in the Mo‐Ga‐C 221 phase.^[^ ^22c^ ^]^ Irregular through‐hole defects are formed in the Mo_1.74_CT_z_ MXene nanosheets as Cr atoms are removed together with Ga during the etching process, as shown in Figure 1b. The AZIBs using a Mo_1.74_CT_z_ MXene flexible self‐standing film as cathode exhibit extraordinary stability during repetitive charging and discharging (up to 100 000 times).

The disappearance of the characteristic X‐ray diffraction (XRD) peaks (around 40°) and the appearance of the 002 peak in Figure 1d indicates that MXene was successfully obtained from the MAX phase alloy. The resulting chemical formula obtained from X‐ray photoelectron spectroscopy is, as shown previously, Mo_1.74±0.06_CO_0.95±0.02_(OH)_0.63±0.01_F_0.3±0.03_.0.2±0.05H_2_O_ads_,^[^ ^22c^ ^]^ the terminations being a result of the top–down wet chemical synthesis. High‐angle annular dark‐field scanning transmission electron microscopy (HAADF‐STEM) imaging of a delaminated MXene after etching demonstrates a thin and transparent nanosheet with sharp edges and lateral size of ∼1 µm (see Figure S1a, Supporting Information). To observe the through‐holes (pores) of the MXene sheet, atomically resolved STEM imaging was used, revealing that defects/holes of various sizes were randomly dispersed across the sheet (Figure 1e; Figures S1b–d, Supporting Information). The holes vary from single atom vacancies to irregularly shaped holes with ∼10 nm diameter. These features can increase the density of active sites and modulate the surface electronic properties of MXenes, positively impacting the performance of electrochemical reactions, such as cycle stability and rate performance. Specifically, the staggered distribution of macropores and atomic‐scale vacancies synergistically enhances stress relief in the electrode during the charging and discharging process, thereby improving the cycle stability of the AZIBs. The cross‐sectional SEM image in Figure 1f shows that a MXene film with a thickness of 1.7 µm possesses a typical lamellar structure. The optical image in the inset demonstrates that the MXene films can be easily wrapped around a rod, demonstrating superior flexibility.

**Figure 2:**
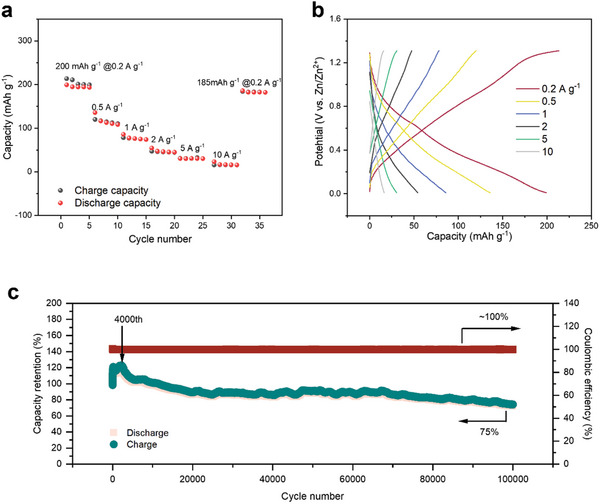
Rate performance and stability of assembled Mo_1.74_CT_z_//Zn AZIBs. a) Rate performance of Mo_1.74_CT_z_//Zn AZIBs at different current densities. b) GCD of Mo_1.74_CT_z_//Zn AZIBs at different current densities. c) Stability of Mo_1.74_CT_z_//Zn AZIBs at 10 A g^−1^.

High performance is a pivotal indicator for potential commercial application of batteries. The defective structure in MXene nanosheets, with more exposed edge active sites and 3D vertical‐short ion channels, is vital to facilitate ion access and accelerate charge transfer at the electrode/electrolyte interface. The Mo_1.74_CT_z_ MXene exhibited excellent capacity, with specific discharge capacities of 200 mAh g^−1^ at current densities of 0.2 A g^−1^ (**Figure** **2**a), which exceeds the capacity of currently reported pure MXene cathode AZIBs (see summary of previous work in Table S1, Supporting Information). The reversible capacity quickly returns to 185 mA h g^−1^ once the rate is tuned back to 0.2 A g^−1^, demonstrating significant high‐rate tolerance. The galvanostatic charge/discharge (GCD) characteristics at different current densities of Zn//MXene were further investigated. The GCD curves in Figure 2b exhibit a typical symmetrical shape, suggesting excellent reversibility of MXene.

Finally, the most outstanding property of the Zn//MXene battery is its ultra‐long cycling sustainability (Figure 2c). This stability is not accidental but is attributed to the well‐preserved structural stability of Mo_1.74_CT_z_ MXene in a 1 M ZnCl_2_ electrolyte. The Mo_1.74_CT_z_‐based zinc‐ion battery demonstrates a long‐term cycling life with a high capacity retention of 75% and a Coulombic efficiency of nearly 100% after 100 000 cycles at 10.0 A g^−1^ (Figure 2c), which is superior to all recently reported AZIBs (see summary of previous work in Table S2, Supporting Information). While a capacity as high as 400 mAh g^−1^ has been reported for the vanadium‐based cathode AZIB, originating from the diverse range of valence states (ranging from +2 to +5), it suffers from stability issues.^[^ ^24^ ^]^ Even at a current density of 1 A g^−1^, it can still maintain 66.5% of its capacity after 100 000 cycles (Figure S2, Supporting Information). Interestingly, the specific capacity shown herein increases until about the 4000th cycle and then fades slowly (Figure 2c). The initial increase in the capacity is likely due to the gradual activation of the electrode. A dynamic saturation of active vacancies is accessible on repeated cycling, leading to higher specific capacities. After 4000th cycle, the Mo_1.74_CT_z_ MXene cathode may begin to dissolve during cycling process, resulting in the loss of active materials and a gradual decline in capacity. However, the phase structure of Mo_1.74_CT_z_ MXene remains unchanged throughout the cycling process (Figure S3, Supporting Information), which further illustrates the excellent potential of Mo_1.74_CT_z_ MXene as a cathode material for long‐life aqueous zinc‐ion batteries.

**Figure 3:**
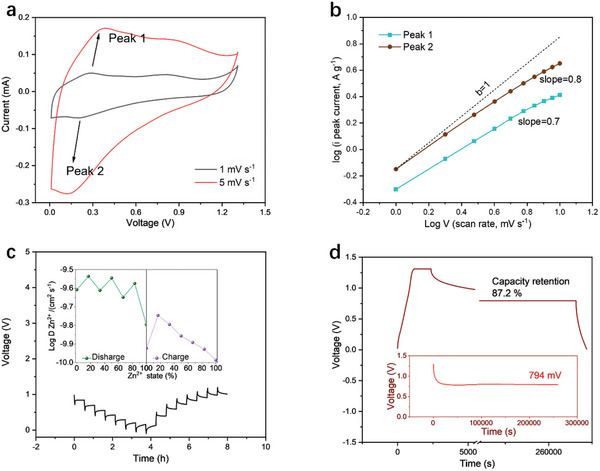
Electrochemical performance of assembled Mo_1.74_CT_z_//Zn AZIBs. a) CV of Mo_1.74_CT_z_//Zn AZIBs at 1 mV s^−1^ and 5 mV s^−1^. b) b‐values of Mo_1.74_CT_z_//Zn AZIBs calculated from anodic and cathodic peaks. c) GITT curve of Mo_1.74_CT_z_//Zn AZIBs at 0.5 A g^−1^. d) Self‐discharge examination combining the GCD and OCV test.

The cyclic voltammogram (CV) curves of the AZIBs were performed in a voltage range of 0.01–1.30 V at scan rates of 1 mV s^−1^ and 5 mV s^−1^ (**Figure** **3**a). As the scan rate increases, the anodic (oxidation peaks) peak shifts to higher potential, while the cathodic peak (reduction peak) gradually shifts to lower potential. It is observed that the capacitive contribution of the Mo_1.74_CT_z_ MXene electrode increases from 49% at 1 mV s^−1^ to 75% at 10 mV s^−1^ (Figures S4, S5, Supporting Information). At the scan rates of 10 mV s^−1^ and higher, the response is essentially controlled by capacitive effects, which are beneficial for achieving excellent cycling stability. The linear fit to the relationship between the logarithm (log) of the peak current and the log of the scan rate (Figure 3b) shows that the slopes of the anodic and cathodic peaks are 0.7 and 0.8, respectively. This demonstrates that the reaction kinetics of MXene are mainly determined by redox reactions rather than ion diffusion. The larger interlayer spacing upon Zn ion insertion and higher electrical conductivity are simultaneously achieved, facilitating the diffusion kinetics of ion insertion/extraction. This is also well reflected in the electrochemical impedance spectroscopy (EIS) curves (Figure S5, Supporting Information), which show that no significant Warburg impedance is observed even after long‐term stability testing.

A galvanostatic intermittent titration technique (GITT) test was carried out to evaluate the diffusion coefficient of Zn^2+^ (D_Zn2+_) in the Mo_1.74_CT_z_ MXene electrode during charge and discharge processes. Figure 3c exhibits the GITT curves of the charge/discharge process of the MXene electrode in the voltage range of 0.01–1.30 V. The calculated D_Zn2+_ values of the Mo_1.74_CT_z_ MXene cathode reached the 10^−9^ to 10^−10^ cm^2^ s^−1^ range (Figure 3c), reflecting the high electrochemical kinetics of the Mo_1.74_CT_z_ MXene cathode in AZIBs.

The self‐discharge phenomenon has been identified as a critical issue for AZIBs. After being fully charged to 1.30 V, the MXene//Zn battery was rested for 72 h to monitor the decay of the open‐circuit voltage, OCV (Figure 3d). As shown in the magnified inset, the OCV of the MXene//Zn battery only decreased from 1.30 to 0.79 V after 72 h, achieving the lowest self‐discharge rate of 7 mV h^−1^. Furthermore, a high proportion of 87.2% capacitance could still be discharged even after the 72‐h rest stage.

**Figure 4:**
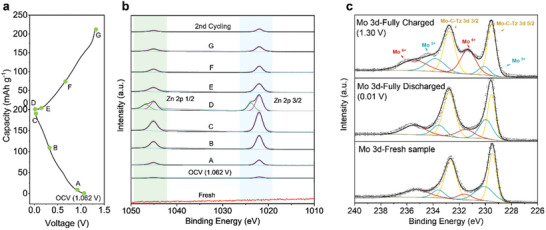
Reversible redox of assembled Mo_1.74_CT_z_//Zn AZIBs. a) Typical discharge/charge curves in the first operation cycle at 0.2 A g^−1^. b) Zn 2p XPS spectra. c) Mo 3d XPS spectra.

To further understand the reaction mechanism of the electrode material, the chemical states of the MXene nanosheets during charge/discharge processes were characterized using XPS photoelectron spectroscopy (**Figure** **4**). As illustrated in Figure 4b, no peaks were observed for Zn species in the Zn 2p region in the pristine MXene electrode, whereas four peaks are observed after discharge to 0.01 V (point D in Figure 4a), demonstrating that zinc ions are inserted into the MXene nanosheets, having different coordinations. After recharging to 1.30 V, two peaks disappear, and the intensity of the other two Zn 2p peaks is much lower than that of the fully discharged state, confirming that most zinc ions are extracted from the electrode. Based on the observed trend in Figure 4b, the appearance/disappearance of the two peaks at 1023.8 and 1046.8 eV should be ascribed to the intercalation of zinc ions into the MXene, while the peaks at 1021.9 and 1045.0 eV could be ascribed to the absorbed Zn^2+^ salts on the electrode surface.^[^ ^10c^ ^]^ The fully charged cathode shows only one Zn 2p3/2 component (1021.9 eV), which is attributed to some remaining surface absorption. The reversible change in peak intensity of Zn 2p also illustrates the adsorption/desorption of Zn^2+^ during the charge and discharge process.

Figure 4c records the evolution of the Mo 3d signal during battery operation. In the pristine electrode, the majority of Mo species have low valence states: (Mo‐C‐T_z_ and Mo +5) forming 87% of the 3d region, and a small fraction is of higher valence state (Mo +6; Figure S7, Supporting Information). When discharged to 0.01 V, the peaks for Mo‐C‐T_z_ and Mo^5+^ rise, showing that Mo^6+^ (3d5/2: 231.5 eV and 3d3/2: 235.3 eV) is reduced to lower valence states (Mo‐C‐T_z_ and +5 valence), corresponding to the change from fully charged state to fully discharged state. Meanwhile, the Zn 2p peaks (2p3/2: 1021.9 eV and 2p1/2: 1045.0 eV) appear. This indicates that Zn ions are successfully inserted into the MXene lattice. Furthermore, it is shown that the Zn‐ion intercalation is reversible. Notably, a small amount of trapped Zn “pillars” in the electrode might serve as interlayers to further reinforce the layered structure of MXene during electrochemical cycles.^[^ ^24^, ^25^ ^]^ Moreover, the interlayer spacing of Mo_1.74_CT_z_ increases further during the cycling process, which promotes the efficient intercalation of zinc ions (Figure S3, Supporting Information).

Altogether, we have obtained a multi‐defect Mo_1.74_CT_z_ MXene from a 3D precursor alloy, (Mo_0.87_Cr_0.13_)_2_Ga_2_C MAX phase, for utilization as electrode material in AZIBs. The MXene displays an excellent stability, indicating that defect engineering is a pathway toward improving stability as well as other properties. The results highlight the potential of MXenes as well as defect engineering for energy storage applications, motivating further exploration of MXenes together with other materials for combining superior stability with further improved energy storage performance.

## 3 Conclusion

In conclusion, we have developed a highly stable MXene cathode, based on multi‐defect Mo_1.74_CT_z_, for a rechargeable aqueous zinc‐ion battery. It achieves 75% capacity retention and nearly 100% Coulombic efficiency after up to 100 000 cycles. The intrinsic structural stability and the large number of vertical holes in the Mo_1.74_CT_z_ MXene alleviate the collapse of the MXene under high current density charge and discharge. The process of zinc ion adsorption/desorption in the MXene and the change in the valence state of Mo are highly reversible, proving the electrochemical stability of the multi‐defect Mo_1.74_CT_z_ cathode. Furthermore, the Mo_1.74_CT_z_‐based AZIBs exhibit good performance with a specific capacity of 200 mA h g^−1^ at a current density of 0.2 A g^−1^, which exceeds the capacity of most MXene‐based AZIBs reported so far. This research provides a new Mo‐based candidate material for stable, low‐flammability, high‐energy‐density, and environmentally friendly AZIBs, taking a step closer to replacing lithium‐ion batteries with ZIBs.

## 4 Experimental Section

### Synthesis of Mo_1.74_CT_z_ MXene Suspension and Mo_1.74_CT_z_ Electrode

A stable Mo_1.74_CT_z_ suspension was obtained through etching of (Mo_0.87_Cr_0.13_)_2_Ga_2_C powder, which was prepared as described in detail elsewhere.^[^ ^22^ ^]^ Typically, 3 g of (Mo_0.87_Cr_0.13_)_2_Ga_2_C powder was slowly added to 50 mL of 25% hydrofluoric acid solution and stirred at 55 °C for 3 days. Afterward, the solid residue was washed several times with deionized water until the pH of the supernatant was above five. The sediments were carefully collected for the next intercalation process.

The intercalation process was realized using 30 mL TBAOH intercalant. TBAOH intercalant was first added to the above‐prepared sediments by shaking it for 10 min in a vortex mixer (LSE, Corning Inc., Glendale, AZ) at a speed of 162 rcf, then the TBAOH was decanted after centrifuging the mixture (6000 rpm for 3 min). The sedimented powder was then washed three times with 40 mL of N_2_‐deaerated deionized (DI) water to completely wash away the remaining TBAOH reagents. The sediment was then dispersed in 40 mL of deionized water by shaking it for 20 min in a vortex mixer. Finally, delaminated Mo_1.74_CT_z_ sheets were obtained after centrifugation at 3000 rpm for 30 min. To obtain the flexible films, the suspension was filtered, and the films were dried naturally for 24 h. These films were punched into small disks of 5 mm diameter and used as electrodes.

### Assembly of Zinc‐Ion Batteries

Bare Zn foil was ultrasonically cleaned with ethanol and subsequently used as a counter electrode for coin cells. The electrolyte was 1 M ZnCl_2_. Glass microfiber (Whatman) was used as a separator. The asymmetric cell was assembled with Mo_1.74_CT_z_ MXene//Zn foil under open atmospheric conditions.

### Material Characterization

The XRD measurement of the MAX phase and MXene was carried out on a PANalytical X'Pert diffractometer using Cu Kα radiation (45 KV and 40 mA). To investigate the defects in the MXene single flakes, high‐angle annular dark‐field (HAADF) scanning transmission electron microscopy (HAADF‐STEM) imaging was performed using Linköping's double‐corrected FEI Titan^3^ (S)TEM electron microscope operated at 300 kV. The cross‐sectional morphology and film thickness were characterized by field emission scanning electron microscopy (Zeiss Sigma 300) equipped with energy dispersive spectroscopy (EDX). XPS measurements were performed on free‐standing delaminated MXene films to evaluate their chemical composition as well as to investigate the chemical nature of the different termination species. The surface analysis system (Kratos AXIS UltraDLD, Manchester, U.K.) comprised monochromatic Al‐Kα (1486.6 eV) radiation with a 45° angle between the sample surface and the X‐ray beam (X‐ray spot was approximately 300 × 800 µm). For XPS fitting, Avantage 5.9931 software was used for fitting, an iterated Shirley method for background correction was applied, and Gaussian–Lorentzian sum functions were used. The binding energy (BE) scale of all XPS spectra was referenced to the Fermi‐edge (EF), which was set to a BE of 0 (zero) eV. The detailed fitting results are shown in Table S3 (Supporting Information).

### Electrochemical Testing

Coin cells were tested at room temperature. The flexible Mo_1.74_CT_z_ MXene was the working electrode. The GCD was probed at different current densities on a NEWARE battery testing system. Cyclic voltammetry and EIS (the frequency was varied from 100 kHz to 0.001 Hz) were evaluated using an electrochemical workstation (VSP, Bio‐Logic, France). The anti‐self‐discharge performance of the Zn‐MXene cell was determined by OCV and GCD tests. Typically, the Zn‐MXene cell was initially fully charged to 1.30V, followed by a 72‐h rest at open‐circuit state to monitor the voltage decay. After this resting stage, the cell was discharged to 0.01 V to calculate the remaining capacitance. GITT measurement was conducted to investigate the dynamic process and equilibrium potential of Mo_1.74_CT_z_ MXene upon discharge and charge using the NEWARE battery testing system.

The specific capacity of the electrode materials was calculated from the CV curves by integrating the charge portion based on the following equation:

(1) $$C=\frac{\int idV}{mv}\;$$

where *i*, *v*, and *m* represent the current, scan rate, and loaded mass of the MXene electrodes, respectively.

The CV curves of Mo_1.74_CT_z_ MXene//Zn ZIB were used to decouple the surface‐controlled and diffusion‐controlled capacitive contributions. The capacitive and diffusion contributions can be calculated using the following equation:

(2) $$i=k_{1}v+k_{2}v^{1/2}$$

where *i* and *v* correspond to the current under the given potential and the scan rate, respectively. The terms *k_1_v* and *k_2_v^1/2^* represent the current delivered by the capacitive process (including the electric double layer and pseudocapacitance) and the diffusion‐controlled Faradaic reaction, respectively.

The slopes of the linear fit during the anodic and cathodic processes were calculated based on the following equation:

(3) $$i=av^{b}$$

where *a* and *b* are adjustable parameters. *i* and *v* represent the peak current and scan rate, respectively. When *b*  =  1, it indicates capacitive charge storage, while *b*  =  0.5 signifies the diffusion‐controlled process.

## Conflict of Interest

The authors declare no conflict of interest.

## Supporting information



Supporting Information

## Data Availability

The data that support the findings of this study are available from the corresponding author upon reasonable request.
