# Supplementary material for: A Hyperstable Aqueous Zinc‐Ion Battery Based on Mo1.74CTz MXene
Source: Small. 2025 Feb 18;21(12):2409122. doi: 10.1002/smll.202409122 (PMC11947507; doi:10.1002/smll.202409122)
Supplement: Supplementary file 1 — Supporting Information [file SMLL-21-2409122-s001.docx]

Supporting Information

A hyperstable aqueous zinc-ion battery based on Mo_1.74_CT_z_ MXene

Ningjun Chen, Rodrigo Ronchi, Joseph Halim, Per O. Å. Persson, Leiqiang Qin*, Johanna Rosen*


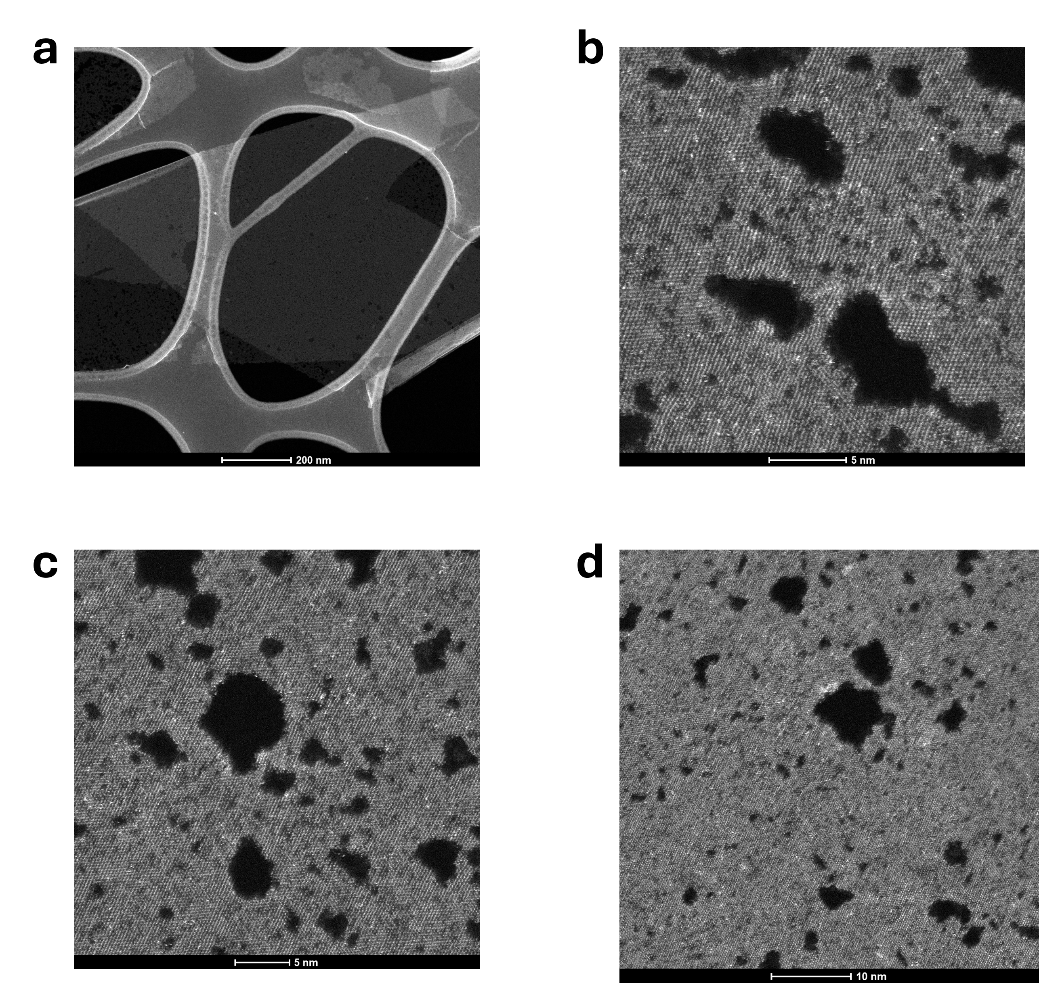


**Figure S1.** TEM of multi-defect Mo_1.74_CT_z_ MXene nanosheets. a) Wing-like morphology of a single Mo_1.74_CT_z_ MXene nanosheet. b-d) Higher magnification TEM-image of Mo_1.74_CT_z_ MXene.

**Table S1**. Capacity comparison of various pure MXene cathodes for AZIBs.

| Materials | Capacity mAh g^-1^ | Current density A g^-1^ | Electrolyte | Ref. |
| --- | --- | --- | --- | --- |
| Mo_1.74_CT_z_ | 200 | 0.2 | 1M ZnCl_2_ | Our work |
| Mo_1.33_CT_x_ | 70 | 1 | 1 M MnSO_4_ | ^[26]^ |
| Ti_3_C_2_T_x_ | 4 | 0.1 | 1 M ZnSO_4_ + 0.05 M MnSO_4_ | ^[27]^ |
| Ti_3_C_2_T_x_ | 20 | 0.2 | 2M ZnSO_4_ | ^[28]^ |
| Mo_1.33_CT_x_ + Ti_3_C_2_T_x_ | 126 | 0.5 | 3 M Zn(CF_3_SO_3_)_2_ | ^[29]^ |
| V_2_CT_x_ | 100 | 0.5 | 21 M LiTFSI + 1 M Zn(CF_3_SO_3_)_2_ | ^[18a]^ |
| Ti_3_C_2_I_2_ | 135 | 0.5 | 2 M ZnCl_2_ + 1 M KCl | ^[30]^ |
| Phenylenediamine-intercalated Ti_3_C_2_T_x_ | 31 | 0.2 | 2M ZnSO_4_ | ^[28]^ |

**Table S2.** Stability comparison of aqueous zinc-ion batteries with different active materials.

| Materials | Capacity retention % | Electrolyte | Ref. | |
| --- | --- | --- | --- | --- |
| Mo_1.74_CT_z_ MXene//Zn | 98.9% after 10 000 cycles,  75% after 100 000 cycles | 1M ZnCl_2_ | | Ourwork |
| Zn_0.3_V_2_O_5_·1.5H_2_O//Zn | 96% after 20 000 cycles | 3 M Zn(CF_3_SO_3_)_2_ | | ^[31]^ |
| Mixed MXene//Zn | 68% after 2000 cycles | 15M ZnCl_2_ | | ^[29]^ |
| CaV_6_O_16_·3H_2_O//Zn | 100% after 10 000 cycles | 4 M Zn (CF_3_SO_3_)_2_ | | ^[32]^ |
| α-MnO_2_&rGO//Zn | 94% after 3000 cycles | 2 m ZnSO_4_ + 0.2 m MnSO_4_ | | ^[33]^ |
| Co_3_O_4_&carbon nanotube fiber//Zn nanosheets | 97.2% after 10 000 cycles | 2 M ZnSO_4_ + 0.0005 M CoSO_4_ | | ^[34]^ |
| CoMn-Prussian blue analogues//Zn | 76.4 % after 1000 cycles | 2M Zn(CF_3_SO_3_)_2_ | | ^[35]^ |
| Mn(BTC)//Zn | 92% after 900 cycles | 2 M ZnSO_4_ + 0.1 M MnSO_4_ | | ^[36]^ |


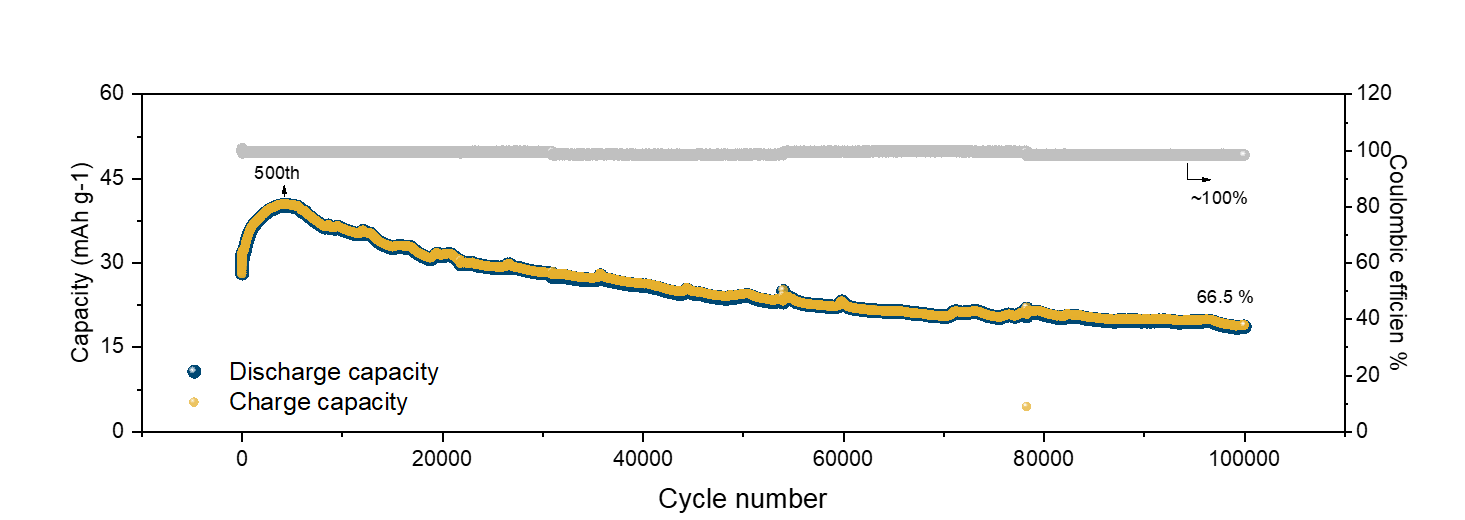


**Figure S2.** Stability of Mo_1.74_CT_z_//Zn AZIBs at 1 A g^-1^.


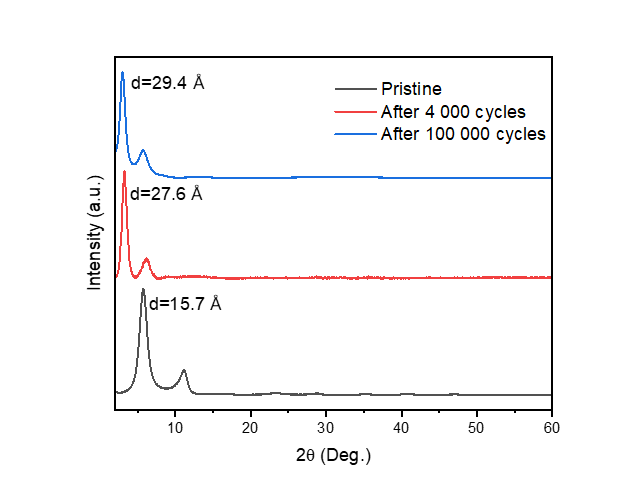


**Figure S3.** The XRD of the fresh Mo_1.74_CT_z_ electrodes compared with after-cycle samples.


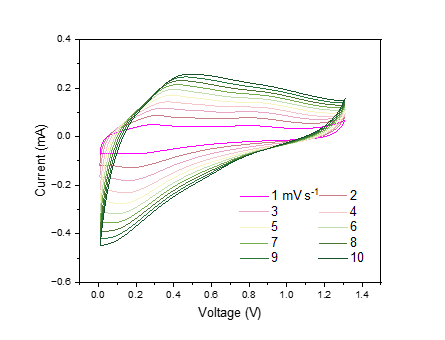


**Figure S4.** CV of Mo_1.74_CT_z_//Zn ZIB at scan rates from 1 to 10 mV s^-1^.


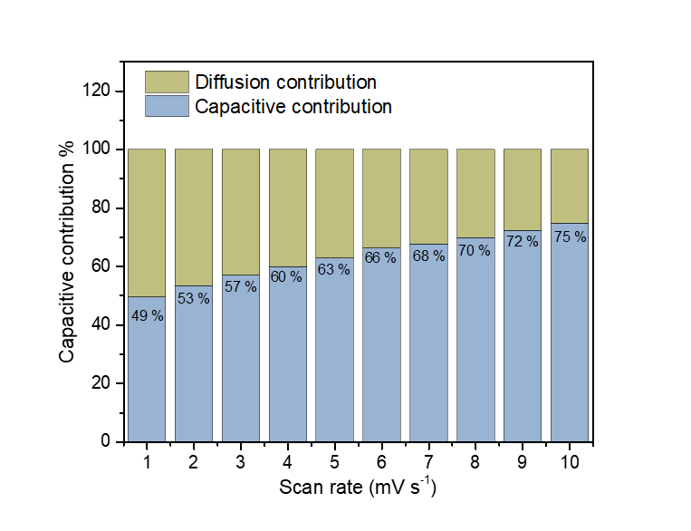


**Figure S5.** Normalized contribution ratio of capacitive capacitance at various scan rates.


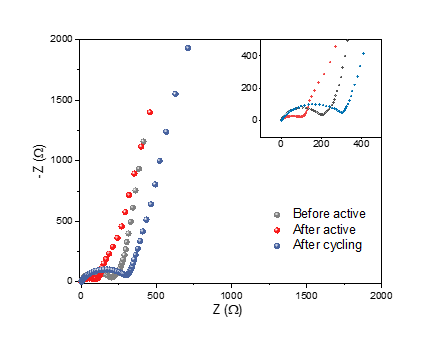


**Figure S6.** EIS of Mo_1.74_CT_z_ //Zn ZIB.


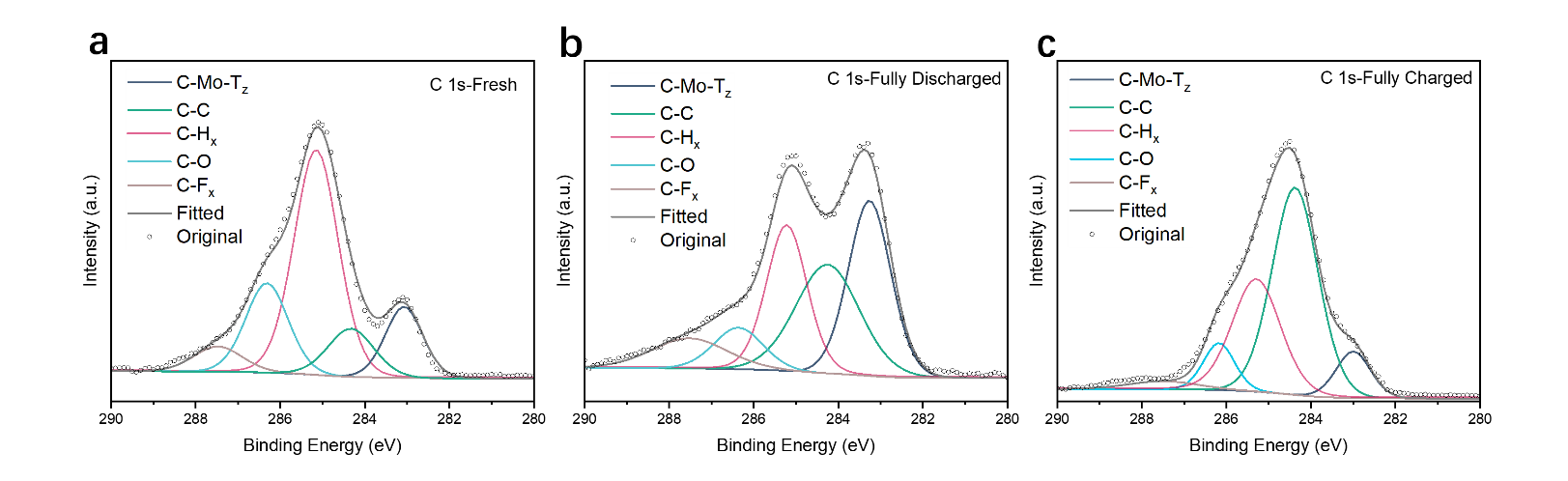
**Figure S7.** C 1s XPS spectra of Mo_1.74_CT_z_ MXene. a) C 1s XPS spectra of fresh sample. b) C 1s XPS spectra of sample after fully discharged. c) C 1s XPS spectra of sample after fully charged.

**Table S 3.** XPS peak fitting results for Mo 3d, C 1s, and Zn 2p regions of Mo_1.74_CT_z_ MXenes before testing, after fully discharged and fully charged.

| Region | Sample | BE [eV] | FWHM [eV] | Fraction | Assigned to | Ref. |
| --- | --- | --- | --- | --- | --- | --- |
| Mo 3d | Mo_1.74_CT_z_  Fresh | 229.3  (232.5)  230.1  (233.5)  231.5  (235.3) | 0.8 (1.3)  1.3 (1.6)  1.3 (1.7) | 0.64  0.23  0.13 | Mo-C-T*_z_*  Mo^+5^  Mo^+6^ | [1-3]  [4]  [1, 4] |
|  | Mo_1.74_CT*_z_*  Fully discharged | 229.3  (232.5)  230.1  (233.5)  231.5  (235.3) | 0.8 (1.2)  1.5 (2.1)  1.1 (1.7) | 0.68  0.25  0.07 | Mo-C-T*_z_*  Mo^+5^  Mo^+6^ | [1-3]  [4]  [1, 4] |
|  | Mo_1.74_CT*_z_*  Fully charged | 229.4  (232.6)  230.0  (233.4)  231.4  (235.2) | 0.9 (1.0)  0.7 (0.8)  0.7 (0.5) | 0.43  0.32  0.25 | Mo-C-T*_z_*  Mo^+5^  Mo^+6^ | [1-3]  [4]  [1, 4] |
| C 1s | Mo_1.74_CT_z_  Fresh | 282.9  284.4  285.1  286.3  287.5 | 1.1  1.3  1.3  1.2  1.4 | 0.16  0.10  0.49  0.20  0.05 | C-Mo-T*_z_*  C-C  CH_x_  C-O  C-F | [1-3]  [3]  [1]  [5]  [6] |
|  | Mo_1.74_CT*_z_*  Fully discharged | 283.0  284.4  285.2  286.4  287.5 | 1.2  1.9  1.2  1.4  2.2 | 0.30  0.28  0.24  0.08  0.10 | C-Mo-T*_z_*  C-C  CH_x_  C-O  C-F | [1-3]  [3]  [1]  [5]  [6] |
|  | Mo_1.74_CT*_z_*  Fully charged | 282.9  284.3  285.1  286.3  287.5 | 1.0  1.3  1.4  0.9  2.0 | 0.11  0.49  0.27  0.11  0.02 | C-Mo-T*_z_*  C-C  CH_x_  C-O  C-F | [1-3]  [3]  [1]  [5]  [6] |
| Zn 2p | Mo_1.74_CT_z_  Fresh | - | - | - | - |  |
|  | Mo_1.74_CT*_z_*  Fully discharged | 1021.9 (1045.0)  1023.8 (1046.8) | 1.9  (1.9)  1.5  (1.7) | 0.46  0.26  0.17  0.11 | Adsorbed Zn^2+^ Intercalated Zn^2+^ | [7, 8]  [9] |
|  | Mo_1.74_CT*_z_*  Fully charged | 1021.9 (1045.0) | 1.7  (1.9) | 0.63  0.37 | Adsorbed Zn^2+^ | [7, 8] |

References

[1] J. Halim, K. M. Cook, P. Eklund, J. Rosen, M. W. Barsoum, *Appl. Surf. Sci.* **2019**, 494, 1138-1147.

[2] Synthesis and Characterization of 2D Molybdenum Carbide (MXene)

[3] R. Ronchi, J. Halim, N. Chen, P. Persson, J. Rosen, *Small Sci.* **2024**, smsc.202400204R1.

[4] X. Guo, C. Wang, , W. Wang, , Q. Zhou, , W. Xu, , P. Zhang, S. Wei, Y. Cao, K. Zhu, Z. Liu, X. Yang, Y. Wang, X. Wu, L. Song, S. Chen, X. Liu, *Nano Res. Energy* **2022**, 1, 9120026.

[5] J. Halim, K. M. Cook, M. Naguib, P. Eklund, Y. Gogotsi, J. Rosen, M. W. Barsoum, *Appl. Surf. Sci.* **2016**, 362, 406-417.

[6] W. Zheng, J. Halim, P. O. Persson, J. Rosen, M. W. Barsoum, *J. Power Sources* **2022**, 525, 231064.

[7] N. Zhang, , M. Jia, , Y. Dong, , Y. Wang, , J. Xu, , Y. Liu, L. Jiao, F. Cheng, *Adv. Funct. Mater.* **2019**, 29(10), 1807331.

[8] X. Wang, Y. Wang, Y. Jiang, X. Li, Y. Liu, H. Xiao, Y. Ma, G. Yuan, *Adv. Funct. Mater.* **2021**, 31(35), 2103210.

[9] X. Z. Zhai, J. Qu, S. M. Hao, Y. Q. Jing, W. Chang, J. Wang, W. Li, Y. Abdelkrim, H. Yuan, Z. Z. Yu, *Nanomicro Lett.* **2020**, 12, 1-15.
